# The Roles of Extracellular Vesicles in Malignant Melanoma

**DOI:** 10.3390/cells10102740

**Published:** 2021-10-14

**Authors:** Ying-Chen Cheng, Yu-An Chang, Yi-Jen Chen, Hsu-Min Sung, Ivan Bogeski, Hong-Lin Su, Ya-Ling Hsu, Hui-Min David Wang

**Affiliations:** 1 Graduate Institute of Biomedical Engineering, National Chung Hsing University, Taichung 402, Taiwan; wizozd51221@gmail.com (Y.-C.C.); s45df62000@gmail.com (Y.-A.C.); 2 Graduate Institute of Clinical Medicine, College of Medicine, Kaohsiung Medical University, Kaohsiung 807, Taiwan; chernkmu@gmail.com (Y.-J.C.); yainghsu@kmu.edu.tw (Y.-L.H.); 3 Department of Physical Medicine and Rehabilitation, School of Medicine, Kaohsiung Medical University, Kaohsiung 807, Taiwan; 4 Department of Physical Medicine and Rehabilitation, Kaohsiung Municipal Siaogang Hospital, Kaohsiung 807, Taiwan; 5 Department of Physical Medicine and Rehabilitation, Kaohsiung Medical University Hospital, Kaohsiung 807, Taiwan; 6 Molecular Physiology, Institute of Cardiovascular Physiology, University Medical Center, Georg August University, 37073 Göttingen, Germany; hsu-min.sung@med.uni-goettingen.de (H.-M.S.); ivan.bogeski@med.uni-goettingen.de (I.B.); 7 Department of Life Sciences, National Chung Hsing University, Taichung 402, Taiwan; suhonglin@nchu.edu.tw; 8 Drug Development and Value Creation Research Center, Kaohsiung Medical University, Kaohsiung 807, Taiwan; 9 Department of Medical Laboratory Science and Biotechnology, China Medical University, Taichung City 404, Taiwan; 10 Graduate Institute of Medicine, College of Medicine, Kaohsiung Medical University, Kaohsiung 807, Taiwan

**Keywords:** extracellular vesicles (EVs), melanoma, angiogenesis, metastasis, invasion, drug resistance, immune system, therapeutic application

## Abstract

Different types of cells, such as endothelial cells, tumor-associated fibroblasts, pericytes, and immune cells, release extracellular vesicles (EVs) in the tumor microenvironment. The components of EVs include proteins, DNA, RNA, and microRNA. One of the most important functions of EVs is the transfer of aforementioned bioactive molecules, which in cancer cells may affect tumor growth, progression, angiogenesis, and metastatic spread. Furthermore, EVs affect the presentation of antigens to immune cells via the transfer of nucleic acids, peptides, and proteins to recipient cells. Recent studies have also explored the potential application of EVs in cancer treatment. This review summarizes the mechanisms by which EVs regulate melanoma development, progression, and their potentials to be applied in therapy. We initially describe vesicle components; discuss their effects on proliferation, anti-melanoma immunity, and drug resistance; and finally focus on the effects of EV-derived microRNAs on melanoma pathobiology. This work aims to facilitate our understanding of the influence of EVs on melanoma biology and initiate ideas for the development of novel therapeutic strategies.

## 1. Melanoma

Human skin is the first layer of defense, protecting us from external factors and providing control of body temperature and storage of moisture and fat. Skin cancer is a common malignancy, with three major types (basal cell carcinoma, squamous cell carcinoma, and melanoma), which have different precursor cells. Basal cell carcinoma and squamous cell carcinoma are classified as non-melanoma skin cancers [1]. Malignant melanoma which is derived from melanocytes is the most aggressive, invasive, and life-threatening skin cancer [2]. Risk factors for melanoma development include fair skin and exposure to sunlight while ultraviolet light exposure is one of the main causes of the development of melanoma [3]. According to the World Health Organization, approximately 132,000 new cases of melanoma are diagnosed each year worldwide. Especially in white (Caucasian) people, melanoma is becoming more common, mostly due to their less pigmented skin, which renders this population more susceptible to ultraviolet light exposure [4].

Metastasis is a multistep process. The first step involves tumor cells invading the basal membrane and entering the blood vessels. Then, these tumor cells circulate in the blood stream until their attachment at the site of metastasis and initiate subsequent extravasation. Finally, they colonize and grow in distal host organs. In melanoma, vascular invasion occurs predominantly via lymphatic vessels. Vascular invasion is associated with factors indicative of a poor prognosis, including stage, increased Breslow thickness, and ulceration [5]. Cells can communicate by different types of EVs, which include exosomes, apoptotic bodies, and microvesicles (MVs) [6]. EVs are important mediators of intercellular communication between cells and distal organs and are crucial to cancer progression [7]. The role of EVs in melanoma remains unclear. Research focusing on understanding the functions and effects of EVs is essential to improve the treatment for melanoma patients and reduce the risk of melanoma metastasis. This review will help scientists understand more about the relationship between EVs and aggressive cancer and inspire in-depth research to make greater progress and breakthroughs.

## 2. Classification and Biology of Extracellular Vesicles

EVs can mediate intercellular communication during many cellular processes, and this role of EVs has piqued the interest of the scientific community. Evidence of the existence and functions of EVs was first collected in 1946 through a combination of ultracentrifugation, electron microscopy, and functional studies [8]. In 1970, the term “extracellular vesicle” was used in a manuscript title for the first time [9]. In the 1970s–1980s, several independent studies identified the release of plasma membrane vesicles from rectal adenoma microvillus cells [10] and discovered virus-like particles in human cell cultures and bovine serum as preliminary findings of exosomes [11]. In 1983, detailed ultrastructural studies indicated that EVs are released through the fusion of multivesicular bodies (MVBs) with the cell membrane during immature red blood cell differentiation [12]. Since 2006, several reports have indicated that nucleic acids, proteins, and other molecules can be transferred between cells via EVs [13]. Through this shuttle-like mechanism, EVs modulate the activity of recipient cells and participate in various physiological and pathological processes, including tumor development, growth, progression, metastasis, and the development of drug resistance [14]. EVs express specific membrane proteins that facilitate EV interactions with particular recipient cells. This process was shown to be involved in organotropic metastatic spread [15]. Following these findings, researchers have isolated EVs from most cell types and biological fluids. The rapid development of the EV research community has been due to the establishment of the International Society of Extracellular Vesicles (ISEV) in the early 2000s, which conducted rigorous and standardized work in this area, including the establishment of the *Journal of Extracellular Vesicles*. Commercial investment in EV diagnosis and treatment has also increased, and many companies have developed several cancer diagnostic tests based on EVs.

**Figure 1:**
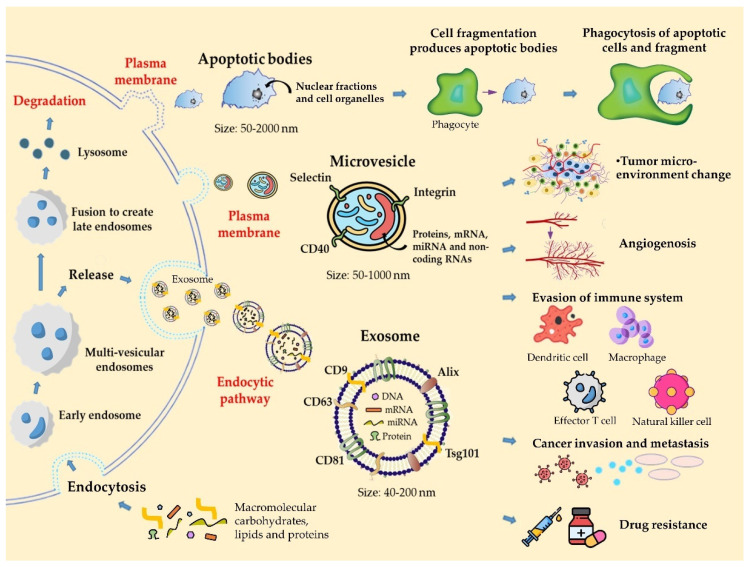
Origin, classification, composition, and biological function of EVs.

Based on their size and biogenesis, EVs can be classified into exosomes, MVs, and apoptotic bodies (Figure 1). Exosomes and MVs can be released by normal cells or cancer cells, although they differ in several aspects. Exosomes are nanosized vesicles of endocytic origin that bud from MVBs toward the lumen of the compartment and are released into the extracellular space. Their size varies from 30 to 100 nm and is limited by the size of the MVBs (40–200 nm) [16]. The content of exosomes includes proteins, DNA, mRNA, and microRNA. In particular, Rab GTPases, soluble N-ethylmaleimide-sensitive factor activating protein receptors (SNAREs), Annexin, and Flotillin are enriched in EVs. Moreover, three transmembrane protein (CD9, CD63, and CD81) families are known to accumulate in the plasma membrane domain and are highly expressed in exosomes, thereby serving as biomarkers for exosomes [17].

EVs are known to facilitate intercellular communication between adjacent cells and distant cells [18,19]. EVs can be released by immune cells as antigen-presenting vesicles to stimulate antitumor immune responses or to induce carcinogenesis via suppressing inflammatory responses. EVs derived from tumor cells have also been shown to promote cancer cell proliferation by inactivating cytotoxic T lymphocytes (CTLs) or natural killer (NK) cells to suppress the immune response and promote regulatory CTL differentiation [20]. Within the nervous system, EVs are thought to be involved in the formation of myelin, the growth of neurites, and the survival of neurons [19]. In addition, several pathogenic proteins, such as viruses and β-amyloid peptides, have been reported to be transferred to other cells via EVs [21]. An important finding is that the roles of mRNAs and miRNAs in EVs from different sources are completely different from each other. Some studies have shown that EVs also circulate in various body fluids, including blood and urine, and their mRNAs and miRNAs can be transferred to recipient cells and participate in many biologically relevant processes, including immune response and angiogenesis [22].

To investigate the characteristics of EVs, a purification protocol is required to be constructed. Early researchers widely used differential ultracentrifugation (DUC) as the method for EV isolation, with its extensive applicability, large capacity, ease of scaling up, and relatively high purification quality. By using DUC, large particles, such as whole cells, cell debris, subcellular structures, and other contaminants, can be removed under a low centrifugal speed. Thenceforth, scientists elevated the centrifugal force to precipitate EVs. Recently, it was verified that DUC outplays five commercial purification kits in terms of vesicle purity, which was a suitable approach for EV research up to the present. However, DUC-isolated EVs may suffer from the contaminants of other non-vesicular particles. Owing to the distinction in particle density between EVs and nano-contamination, density-gradient ultracentrifugation (DGUC) was applied to increase the purity of EVs. According to the minimal information from studies of extracellular vesicles 2018 (MISEV2018), several components, especially sucrose gradient solution, were applied as the medium for EV purification. Configuring the separation medium with gradient densities across the density range of EVs, crude isolated samples are loaded onto the top of the medium and undergo a longer ultracentrifugation period for further purification. Despite all of the advantages of DUC and DGUC, the pricey equipment and time-consuming process are a matter of concern. The centrifugal- and shearing-force-induced structural damage or aggregation also impede the downstream application. Nonetheless, the enhanced purity and quality of EVs provide a step forward in the research on nanoparticles [23].

## 3. EVs Derived from Melanoma and Their Role in Cancer Progression

Biological information between adjacent tumor cells can be transmitted through tumor-derived EVs in a paracrine manner. This signal transduction between malignant cells not only promotes cancer growth and metastasis but also can interfere with normal signaling pathways [24]. Tumor cells may metastasize to distant organs in the body and regulate the tumor microenvironment to form pre-metastatic niches; in these cases, tumor-derived EVs may be potential biomarkers for tumor progression and invasion [25]. In addition, tumor-derived EVs are expected to be used as carriers for cell-free vaccines and for the delivery of specific tumor therapeutic molecules. In this section, we focus on the role of tumor-derived EVs in melanoma development and metastasis and their potential applications in advancing the diagnosis and treatment of melanoma and personalized medicine.

### 3.1. Growth and Angiogenesis

The literature indicates that the addition of EVs to a human cell culture enhances EV production and supports cell proliferation [20]. The biodistribution of cancer-derived EVs in tumor tissues is an important factor in determining the role of EVs in tumor proliferation [26]. In vivo experiments have shown that B16BL6 melanoma cells secrete and absorb B16BL6 cell-secreted EVs to induce their own proliferation and inhibit their own apoptosis, promoting tumor progression [27]. EV uptake by target cells relies on the integrity of plasma membrane microdomains, namely lipid rafts, which are known to be enriched with cholesterol. Scavenger receptor type-B1 (SR-B1) is a high-affinity receptor for mature high-density lipoproteins (HDLs), and SR-B1 maintains cholesterol equilibrium, uptakes extracellular material, and promotes cell signaling [28,29]. The expression of SR-B1 in melanoma enhances EV formation and cellular uptake, promoting a metastatic phenotype. SR-B1 is associated with the expression of microphthalmia-associated transcription factor (MITF) and the regulation of proto-oncogene mesenchymal-to-epithelial transition (MET) factor. SR-B1 is a key molecule for regulating EV uptake and cancer growth [30]. Wnt Family Member 5A (WNT5A) regulates the release of EVs containing the immunomodulatory cytokine IL-6 and proangiogenic factors IL-8, VEGF, and MMP2 from melanoma cells (MeWo, SKmel28, A2058, A375, and HTB63). This effect increases angiogenic processes and facilitates metastatic spread [31]. Hood et al. indicated that melanoma EVs can boost endothelial angiogenic responses to create a premetastatic niche [32]. A previous report indicated that miR-155 in melanoma-derived EVs can induce reprogramming of fibroblasts into CAFs (cancer-associate fibroblast) and trigger the proangiogenic switch of these CAFs [33].

### 3.2. Migration and Invasion

Studies on melanoma cell migration and invasion and on the underlying molecular mechanisms are essential for improving melanoma diagnosis, prognosis, and therapy. EVs play an important role in this regard and regulate the migratory and invasive capacity of melanoma cells. Several studies have demonstrated that EVs can increase migratory and invasive capacities [34]. EVs derived from melanoma cells have also been shown to increase type I interferon receptor (IFNAR1) and cholesterol 25-hydroxylase (CH25H) in normal cells, thus facilitating EV uptake and pre-metastatic niche development [35]. Matrix metalloproteinases (ADAM) and ADAM with thrombospondin motifs (ADAMTS) are enriched in melanoma-derived EVs. These proteins are critical for degrading the extracellular matrix of cancer cells and increasing metastatic spread [36]. Insulin-like growth factor 2 mRNA-binding protein 1 (IGF2BP1) is a multifunctional RNA-binding protein that has been linked to the development of a variety of malignancies. According to previous research, EVs derived from IGF2BP1-overexpressing melanoma cells exacerbate EV-induced metastasis [37]. Xiao et al. showed an increase in invasiveness when normal melanocytes were treated with melanoma EVs [38]. Melanoma usually metastasizes to the lungs, bones, liver, and brain and rarely to other organs. The current mechanism of this pattern needs further understanding, but it is likely that EVs play an important role. For example, melanoma cells are exposed to bone-derived soluble factors, which are related to the molecular activation pathway of stromal-cell-derived factor 1 (SDF-1)/CXC chemokine receptor type 4 (CXCR4)/type 7 CXC chemokine receptor (CXCR7). To this end, EVs reprogram the innate osteotropism of melanoma cells by upregulating their CXCR7 expression [39]. These results suggest that melanoma-cell-derived EVs contribute to melanoma metastasis. In addition, adipocytes secrete EVs, which are oxidized by fatty acids and are absorbed by tumor cells, resulting in increased metastasis and invasion of melanoma [40]. EVs from melanoma cells with poor metastatic potential potently inhibit metastasis to the lung and trigger immune surveillance, resulting in the elicitation of a broad range of monocyte (PMO)-reliant innate immune responses. Furthermore, Plebanek et al. suggested that cancerous cells are cleared at the pre-metastatic niche [41].

### 3.3. Tumor Microenvironment

The interactions of cancer cells with their environment determines whether the primary tumor is contained, metastasizes, or establishes dormant micrometastases. EVs play essential roles in the interstitial transport and intercellular communication within the tumor microenvironment (TME). Metastatic tumor cells show increased ability to sort EV cargo (i.e., proteins and microRNAs) and to release EVs. EV cargo is then transferred to stromal cells, including those that are present in premetastatic niches. Furthermore, EVs promote tumorigenesis and invasion through a variety of mechanisms, resulting in premetastatic niche formation. The following table describes the roles of EVs in the TME [42] (Table 1).

### 3.4. Immune System

The tumor microenvironment controls immune surveillance and anti-tumor immunity [49], mainly through intra- and extracellular signaling. Immunoediting is a complex process that includes intra- and extracellular signals. EVs play an important role in immune escape, both directly and indirectly. The direct modulation of either immune cells or their immature precursors is mostly driven by EV-mediated anti-apoptotic or pro-apoptotic signaling during the melanoma cell migration. The indirect roles of EVs include the expansion and differentiation of negative regulators of the immune system, such as myeloid-derived suppressor cells (MDSCs) and regulatory T lymphocytes (Tregs), thus promoting tumor cell escape from immune surveillance [50,51]. Several effects, i.e., mechanisms link EVs and the immune system (Table 2). Studies have shown that EVs secreted by tumor cells protect and maintain the growth of cancer cells, while EVs produced by normal cells, especially stem cells, inhibit tumor growth and suppress cancer progression [52]. Homing of melanoma exosomes to sentinel lymph nodes imposes synchronized molecular signals that effect melanoma cell recruitment, extracellular matrix deposition, and vascular proliferation in the lymph nodes [53]. In addition, tumor-derived EVs were shown to interfere with immunization by inducing loss of antigen expression, suppression of immune effector cells, exchange of nucleic acids, changes in recipient cell transcription, and inhibition of the immune cell response [54]. Other studies point out that tumor cells and tumor-infiltrating immune cells form a highly tolerant microenvironment, increasing tumor growth and allowing metastatic spread. Studies of anti-tumor immunity have explored the host’s immune responses and promote the development of new therapies and novel methods for use in future therapeutic methods [55].

### 3.5. Drug Resistance and Clinical Treatment

EVs are involved in the development and regulation of different cancer-related processes. Drug resistance of cancer cells is a huge clinical problem and requires further investigation. Nevertheless, it is known that drug-resistant tumor cells are able to enclose chemotherapeutic agents in EVs and transfer anticancer drugs out of tumor cells. Therefore, understanding the molecular mechanisms and signaling pathways of EV-mediated drug resistance will help in the design of novel cancer treatments.

A large number of studies indicate that EVs play a crucial role in the development of the drug resistance of cancer cells (Table 3). Previous research has indicated that the use of BRAF kinase inhibitors (vemurafenib and dabrafenib) to treat melanoma patients bearing the BRAF-activating mutation V600E results in tumor regression, followed by quick development of drug resistance. Receptor tyrosine kinases (RTKs) are upregulated and activate the PI3K-Akt signaling pathway. EVs from drug-resistant melanoma cells were enriched with the RTK PDGFRβ, and delivering EVs rich in PDGFRβ to metastatic melanoma cells with the BRAF inhibitor-sensitive phenotype activated the PI3K/AKT pathway and resulted in the development of drug resistance [64]. Moreover, a novel truncated form of anaplastic lymphoma kinase (ALK) named ALK^RES^ was found to be secreted in EVs. The transfer of ALK^RES^ to sensitive, ALK-negative melanoma cells caused activation of the MAPK signaling pathway and transferred the characteristics of drug resistance to the recipient cells [65].

### 3.6. Small RNA (microRNA)

MicroRNAs (miRNAs) constitute a class of small single-stranded noncoding RNAs (~22 nt in length) that suppress gene expression. miRNAs are transcribed in the nucleus by RNA polymerase II or III. Primary miRNA transcripts (pri-miRNAs) are cleaved through a complex that consists of the endonuclease Drosha and the RNA-binding protein DGCR8. Hairpin pre-miRNAs are exported to the cytoplasm and are cleaved by the endonuclease Dicer to form dsRNA–miRNA duplexes. The complementary strand of the mature miRNA sequence is degraded, facilitating miRNA-induced silencing complex (RISC) formation and targeting the complementary sequences in the 3′ UTR of target mRNAs inhibit translation. miRNAs regulate many physiological and pathophysiological processes, such as growth, differentiation, and cancer progression. miRNAs regulate hundreds of genes; thus, miRNAs can cause complex phenotypic changes [66]. The loss of certain miRNAs facilitates cancer growth, whereas overexpression of other miRNAs promotes cancer progression [67]. miRNAs change the phenotype of melanoma cells and metabolic pathways during melanoma progression. They also affect the extracellular matrix (ECM), which includes fibroblasts, endothelial cells, and immune system cells [68]. miRNAs have different functions in each step of the development of different cancers [69]. Cells have the ability to selectively sort miRNAs into EVs for secretion to nearby or distant targets. Moreover, certain disease states have also identified dysregulated EV-miRNA content, shedding light on the potential role of selective sorting in pathogenesis. The latest findings regarding the roles of EVs-relevant miRNAs in melanoma pathobiology are summarized in Table 4.

## 4. Therapeutic Applications of Extracellular Vesicles

There are several studies on EVs in therapeutic applications. Interestingly, tumor cells release subpopulations of EVs that differ in their molecular and biological characteristics. These differences are essential for the precise transfer of biological information between cells. Accordingly, different components of EVs derived from different cells have different effects depending on their source. Based on these features, monitoring EV phenotypes during treatment enables the discovery of specific EV profiles and an understanding of how these correlate with drug resistance development in melanoma patients. Further analysis of EV heterogeneity will help in understanding the biology of EVs in health and disease and accelerate the development of EV-based diagnostic and therapeutic approaches. Melanoma is diagnosed with cancer-specific EV phenotypes from melanoma patient plasma by a multiplex EV phenotype analyzer chip that incorporates a nano-mixing-enhanced microchip and a multiplex surface-enhanced Raman scattering (SERS) nanotag system [82]. For these reasons, the therapeutic potential of EVs deserves further consideration in the context of drug delivery and regenerative medicine [83]. For example, EVs combined with liposomes and nanoparticles offer novel therapeutic delivery methods. Specifically, EVs derived from cancer cells can be carriers of drugs for delivery and can effectively inhibit tumor proliferation because of their ability to transfer biologically active components and overcome biological barriers [84]. In addition to being considered as potential therapeutics, EVs have the ability to enhance tissue regeneration and serve as potential replacements for stem cell therapy, playing a role in reimmunization, which promotes regeneration and inhibits pathogens [85]. These properties can lead to a wide range of therapeutic applications, including vaccination, treatment for autoimmune diseases, cancer, and tissue damage (Table 5). In recent years, many drugs for melanoma have been developed, but stimulating cancer cell death is still the major strategy [86]. If cancer cells acquire drug resistance, therapeutic treatment becomes challenging and the mortality rate significantly increases. The injected EVs derived from colon cancer through the tail vein of NOD.CB17-Prkdc^scid^/NcrCrlBltw mice determined neoplastic transformation and metastases in the lungs of the mice [87]. Another study proved that the timing of EV administration is as critical as that of oral administration after resection of the primary tumor reversed the pro-metastatic effects of milk-derived EVs in breast cancer models [88]. EVs from a highly metastatic clonal variant of the osteosarcoma cell line were internalized by a poorly metastatic clonal variant of the same cell line and induced a migratory and invasive phenotype. It was pointed out that EVs derived from highly metastatic clonal variants drive metastatic behaviors [89]. EVs originated from the brain carry messages to cancer cells that modify glioma cell metabolism, reducing lactate, nitric oxide (NO), and glutamate (Glu) release. EVs affect Glu homeostasis, increasing the expression of Glu transporter Glt-1 on astrocytes [90]. Recently, there is increasing evidence showing that EVs promote cancer progression and metastasis. It is suggested that clinicians effectively control the secretion of pernicious exosomes and melanoma will be remedied comprehensively.

## 5. Conclusions

**Figure 2:**
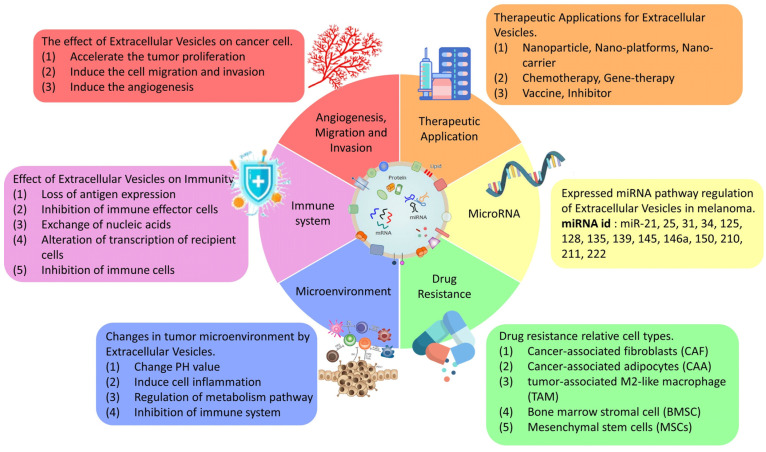
The summary of EV effectiveness in cancer therapeutic treatments.

EVs are important modulators of inter- and intracellular communications. EVs regulate diverse cellular processes, and they also contribute to cancer development and metastasis. The EV components can be transferred to other cells, affecting the physiological processes of the recipient cells and influencing the entire tumor microenvironment. EVs offer a valuable alternative to the current therapeutic options. They serve as nanoscale vehicles for drug delivery and have great potentials in this regard, not only because of their high biocompatibility, but also due to their low cytotoxicity. Tumor-cell-derived extracellular vesicle surface antigens have a huge effect on the immune system and can be modified by various agents to directly affect tumor cells or regulate antitumor immunity. The high EV heterogeneity is a problem in exploring their full therapeutic potential. Regarding their application as drug carriers, the disadvantages include low transfection efficiency and high dependence on cell division for cases in which cell cycle manipulation is required. In addition, the experimental conditions must be precisely controlled during nanoparticle delivery to avoid vesicle rupture. Thus, verifying the roles of EVs in clinical practice is not a simple endeavor, and more research is needed before EVs can be practically applied as therapeutic tools (Figure 2). Nevertheless, EVs offer a fully new approach for treating melanoma and other cancers. Understanding their regulation and biological features has a high potential to improve cancer treatment in the future.

## Figures and Tables

**Table 1:** The mechanisms of tumor microenvironment regulations in cancers.

Method	Mechanism	Reference
pH	Extracellular acidity may increase the ability of cancer cells to release EVs. The pH of the environment can be used to regulate the release of EVs, affecting the development of the tumor or the control of drug resistance.	[43]
EMT pathway	During EV-mediated epithelial–mesenchymal transition (EMT)-like processes, the mitogen-activated protein kinase (MAPK) signaling pathway is activated and promotes metastasis. It was demonstrated that melanoma-cell-derived EVs promote the EMT in the tumor microenvironment.	[44]
Inflammatory	EVs secreted by metastatic melanoma cells spontaneously metastasize to the lungs and brain and activate proinflammatory signals that induce cell inflammation to promote tumor metastasis.	[45]
Metabolism	miRNA inhibitors of melanoma-derived EVs regulate stromal cell metabolism, inhibit the activity of miR-155 and miR-210, and may contribute to the promotion of metastasis.	[46]
Immune system	The lipid, protein, DNA, mRNA, and miRNA components in EVs are transferred to recipient tumor cells, affecting many immune-related pathways, leading to the activation, differentiation, and expression of the immune cells and the regulation of the tumor microenvironment, thus affecting tumor development, metastasis, and drug resistance. EVs are regulated and released by the TME and regulate the cell biology of myeloid-derived suppressor cells (MDSCs), including promoting their activation and amplification and enhancing their immunosuppressive functions.	[47,48]

**Table 2:** The effect of tumor-derived EVs in immune systems.

Target	Mechanism	Reference
CD8(+) effector T cells	Melanoma-derived EVs induce immune suppression by promoting T regulatory cell expansion and destroying antitumor CD8(+) effector T cells, thus leading to tumor escape.	[56]
CD4+ T cells	Melanoma-derived EVs may directly activate the mitochondrial apoptotic pathway of CD4+ T cells through the microRNA in the EVs.	[57]
PTEN	Tumor-secreted miR-214 is sufficiently delivered to recipient T cells by EVs specifically targeting mouse peripheral CD4+ T cells. miR-214 downregulates phosphatase and tensin homolog (PTEN) and promotes Treg expansion. Tumor-derived EVs enhance immune suppression and tumor implantation/growth in mice.	[58]
MHC	The major histocompatibility complex (MHC) class I molecules and EVs have an important correlation with the induction of antigen-specific T cell responses and the stable development of tumors.	[59]
PD-L1	Increased tumor surface expression of programmed death-ligand 1 (PD-L1) facilitates tumor cell escape from immune surveillance. PD-L1 interacts with the programmed death-1 (PD-1) receptor on T cells to elicit the immune checkpoint response. Metastatic melanomas release EVs that carry PD-L1 on their surface, which suppresses the function of CD8(+) T cells and facilitates tumor growth.	[60]
PTPN11	Melanoma-derived EVs provide a complex biological load, and the upregulation of tumor tyrosine-protein phosphatase nonreceptor type 11 (PTPN11) expression by B16F0 EVs suppresses T lymphocyte function.	[61]
M1 and M2 macrophages	EVs derived from melanoma in premetastatic lymph nodes trigger angiogenesis in tumors by inducing classically activated (M1) and alternatively activate (M2) macrophage-mediated angiogenesis by inducing endothelial cell proliferation.	[62]
NKG2D	Melanoma-cell-derived EVs downregulate NKG2D expression in natural killer cells to induce immune suppression.	[63]

**Table 3:** The effect of tumor-derived EVs on drug resistance.

Gene ID	Mechanisms	Reference
*ALK*	ALK activates the MAPK signaling pathway to target cancer. Combined treatment with the inhibitor of ALK and BRAF can significantly reduce tumor growth and induce apoptosis in melanoma.	[65]
*PDGFRβ*	PDGFR*β* is a resistance driver transferred to recipient melanoma cells via EVs, resulting in the activation of phosphoinositide 3-kinases (PI3K)/protein kinase B (PKB) signaling and escape from the MAPK pathway in BRAF-inhibitor-sensitive cells, thus influencing drug sensitivity in the recipient melanoma cells.	[64]

**Table 4:** The mechanisms and target locations of microRNA in melanoma.

miRNA ID	EV Origin	Effect	Target Site	Reference
let-7g-5p	Patient’s plasma	Increases levels of let-7g-5p in EVs, which is associated with better disease control	MAPK	[70]
miR-34a	Patient’s plasma	Prevents tumor relapse and blocks tumor cell proliferation	*β*-catenin	[71]
miR-211	Melanosome	Targets IGF2R and leads to activation of MAPK signaling, which promotes melanoma growth	IGF2R	[72]
miR-222	Melanoma EVs	Increases tumor malignancy	PI3K/AKT	[73]
miR-155, miR-210	Melanoma EVs	Modulate stromal cell metabolism, which promotes the development of metastasis	OXPHOS	[46]
miR-709, miR-2137	Melanoma EVs	Modulate T cell function	PD-L1	[74]
miR-494	Melanoma EVs	Suppresses tumor growth and metastasis when levels are increased	none	[75]
miR-146a, miR-155, miR-125b, miR-100, miR-125a, miR-146b, miR-99b	Melanoma EVs	Convert myeloid cells into myeloid-derived suppressor cells	CTLA-4, PD-1	[76]
miR-106b-5p	Melanoma EVs	Activates the ERK pathway	EphA4	[77]
miR-205	Melanoma	Regulates E2F-regulated AKT phosphorylation to inhibit the proliferative capacity of melanoma cells	E2F1, E2F5	[78]
miR-182	Melanoma	Suppresses the expression of MITF and FOXO3 and stimulates migration of melanoma cells	MITF and FOXO3	[79]
miR-21	Melanoma	Upon upregulation in melanocytes, increases the proliferation rate and decreases the apoptosis rate	PTEN	[80]
miRNA-342	Melanoma	Targets zinc-finger E-box-binding homeobox 1 (ZEB1) and decreases the proliferation and invasion rates of melanoma cells.	ZEB1	[81]

**Table 5:** The therapeutic applications of EVs in cancer.

Method	Mechanisms	Reference
Nanoparticle	Acridine orange (AO) is an eosinophilic dye that is coated onto a system with EVs as nanocarriers for molecular therapy. AO not only extends the time of drug delivery but also attenuates the toxicity induced in normal cells. Exo-AO treatment has great potential and can be used as a new method for treating tumors by delivering Exo-AO. Nanoplatforms, such as EVs modified with targeting ligands, can improve the anticancer and anti-inflammatory effects of imperialin. The system not only significantly improves the release of the drug in the tumor but also is more biocompatible, showing extremely low systemic toxicity both in vitro and in vivo. This platform provides a new method for more efficient use of EVs for drug delivery and targeting. EV biomimetic porous sputum nanoparticles (PSiNPs) secreted by biocompatible tumor cells were developed as drug carriers for targeting cancer chemotherapy. After intravenous administration, the drug is delivered with specificity.	[91,92,93]
Chemotherapy	EVs can act as carriers for chemotherapeutic/chemopreventive agents to suppress tumor proliferation.	[94]
Vaccine	EVs loaded with tumor antigens and Mycobacterium tuberculosis antigens have great potential to be used as vaccines to overcome the immune escape of tumor cells after genetic modification.	[95]
Gene therapy	The suicide fusion gene construct was loaded into EVs derived from nontumorigenic cell lines. Delivery to glioblastoma cell lines and spheres effectively induced apoptosis of glioblastoma cells and thus inhibited tumor growth in vivo.	[96]
Inhibitor	CD133 (Prominin-1) is a stem cell marker that is involved in the development of tumors, differentiation, and anticancer treatment. The use of histone deacetylase 6 (HDAC6) inhibitors to induce CD133 + release in cancer cell EVs has potential as an antitumor mechanism.	[97]

## Data Availability

Not applicable.

## References

[1] Hogue L., Harvey V.M. (2019). Basal cell carcinoma, squamous cell carcinoma, and cutaneous melanoma in skin of color patients. Dermatol. Clin..

[2] Bandarchi B., Ma L., Navab R., Seth A., Rasty G. (2010). From Melanocyte to Metastatic Malignant Melanoma. Dermatol. Res. Pract..

[3] Volkovova K., Bilanicova D., Bartonova A., Letašiová S., Dusinska M. (2012). Associations between environmental factors and incidence of cutaneous melanoma. Review. Env. Health-Glob..

[4] Elder D.E., Bastian B.C., Cree I.A., Massi D., Scolyer R.A. (2020). The 2018 World Health Organization classification of cutaneous, mucosal, and uveal melanoma: Detailed analysis of 9 distinct subtypes defined by their evolutionary pathway. Arch. Pathol. Lab. Med..

[5] Storr S.J., Safuan S., Mitra A., Elliott F., Walker C., Vasko M.J., Ho B., Cook M., Mohammed R.A., Patel P.M. (2012). Objective assessment of blood and lymphatic vessel invasion and association with macrophage infiltration in cutaneous melanoma. Mod. Pathol..

[6] Steinbichler T.B., Dudás J., Riechelmann H., Skvortsova I.-I. (2017). The role of exosomes in cancer metastasis. Semin. Cancer Biol..

[7] Gowda R., Robertson B.M., Iyer S., Barry J., Dinavahi S.S., Robertson G.P. (2020). The role of exosomes in metastasis and progression of melanoma. Cancer Treat Rev..

[8] Yáñez-Mó M., Siljander P.R., Andreu Z., Zavec A.B., Borràs F.E., Buzas E.I., Buzas K., Casal E., Cappello F., Carvalho J. (2015). Biological properties of extracellular vesicles and their physiological functions. J. Extracell Vesicles.

[9] Witwer K.W., Théry C. (2019). Extracellular vesicles or exosomes? On primacy, precision, and popularity influencing a choice of nomenclature. J. Extracell Vesicles.

[10] De Broe M., Wieme R., Roels F. (1975). Letter: Membrane fragments with koinozymic properties released from villous adenoma of the rectum. Lancet.

[11] Benz E.W., Moses H.L. (1974). Small, virus-like particles detected in bovine sera by electron microscopy. J. Natl. Cancer Inst..

[12] Pan B.T., Johnstone R.M. (1983). Fate of the transferrin receptor during maturation of sheep reticulocytes in vitro: Selective externalization of the receptor. Cell.

[13] Valadi H., Ekström K., Bossios A., Sjöstrand M., Lee J.J., Lötvall J.O. (2007). Exosome-mediated transfer of mRNAs and microRNAs is a novel mechanism of genetic exchange between cells. Nat. Cell Biol..

[14] Santos P., Almeida F. (2020). Role of Exosomal miRNAs and the Tumor Microenvironment in Drug Resistance. Cells.

[15] Hoshino A., Costa-Silva B., Shen T.L., Rodrigues G., Hashimoto A., Tesic Mark M., Molina H., Kohsaka S., Di Giannatale A., Ceder S. (2015). Tumour exosome integrins determine organotropic metastasis. Nature.

[16] Coscia C., Parolini I., Sanchez M., Biffoni M., Boussadia Z., Zanetti C., Fiani M.L., Sargiacomo M. (2016). Generation, Quantification, and Tracing of Metabolically Labeled Fluorescent Exosomes. Methods Mol. Biol..

[17] Surman M., Stępień E., Przybyło M. (2019). Melanoma-Derived Extracellular Vesicles: Focus on Their Proteome. Proteomes.

[18] Desrochers L.M., Bordeleau F., Reinhart-King C.A., Cerione R.A., Antonyak M.A. (2016). Microvesicles provide a mechanism for intercellular communication by embryonic stem cells during embryo implantation. Nat. Commun..

[19] Raposo G., Stoorvogel W. (2013). Extracellular vesicles: Exosomes, microvesicles, and friends. J. Cell Biol..

[20] Hood J.L. (2019). Natural melanoma-derived extracellular vesicles. Semin. Cancer Biol..

[21] Pegtel D.M., Peferoen L., Amor S. (2014). Extracellular vesicles as modulators of cell-to-cell communication in the healthy and diseased brain. Philos. Trans. R Soc. Lond. B Biol. Sci..

[22] Yang Q., Diamond M.P., Al-Hendy A. (2016). The emerging role of extracellular vesicle-derived miRNAs: Implication in cancer progression and stem cell related diseases. J. Clin. Epigenet.

[23] Dong L., Zieren R.C., Horie K., Kim C.J., Mallick E., Jing Y., Feng M., Kuczler M.D., Green J., Amend S.R. (2020). Comprehensive evaluation of methods for small extracellular vesicles separation from human plasma, urine and cell culture medium. J. Extracell. Vesicles.

[24] Becker A., Thakur B.K., Weiss J.M., Kim H.S., Peinado H., Lyden D. (2016). Extracellular Vesicles in Cancer: Cell-to-Cell Mediators of Metastasis. Cancer Cell.

[25] Alipoor S.D., Mortaz E., Varahram M., Movassaghi M., Kraneveld A.D., Garssen J., Adcock I.M. (2018). The potential biomarkers and immunological effects of tumor-derived exosomes in lung cancer. Front. Immunol..

[26] Lee S.S., Won J.H., Lim G.J., Han J., Lee J.Y., Cho K.O., Bae Y.K. (2019). A novel population of extracellular vesicles smaller than exosomes promotes cell proliferation. Cell Commun. Signal.

[27] Matsumoto A., Takahashi Y., Nishikawa M., Sano K., Morishita M., Charoenviriyakul C., Saji H., Takakura Y. (2017). Accelerated growth of B16BL6 tumor in mice through efficient uptake of their own exosomes by B16BL6 cells. Cancer Sci..

[28] Valacchi G., Sticozzi C., Lim Y., Pecorelli A. (2011). Scavenger receptor class B type I: A multifunctional receptor. Ann. N.Y. Acad. Sci..

[29] Simons K., Vaz W.L. (2004). Model systems, lipid rafts, and cell membranes. Annu. Rev. Biophys. Biomol. Struct..

[30] Kinslechner K., Schütz B., Pistek M., Rapolter P., Weitzenböck H.P., Hundsberger H., Mikulits W., Grillari J., Röhrl C., Hengstschläger M. (2019). Loss of SR-BI Down-Regulates MITF and Suppresses Extracellular Vesicle Release in Human Melanoma. Int. J. Mol. Sci..

[31] Ekström E.J., Bergenfelz C., von Bülow V., Serifler F., Carlemalm E., Jönsson G., Andersson T., Leandersson K. (2014). WNT5A induces release of exosomes containing pro-angiogenic and immunosuppressive factors from malignant melanoma cells. Mol. Cancer.

[32] Hood J.L., Pan H., Lanza G.M., Wickline S.A. (2009). Consortium for Translational Research in Advanced, I.; Nanomedicine, Paracrine induction of endothelium by tumor exosomes. Lab. Invest..

[33] Zhou X., Yan T., Huang C., Xu Z., Wang L., Jiang E., Wang H., Chen Y., Liu K., Shao Z. (2018). Melanoma cell-secreted exosomal miR-155-5p induce proangiogenic switch of cancer-associated fibroblasts via SOCS1/JAK2/STAT3 signaling pathway. J. Exp. Clin. Cancer Res..

[34] Kramer N., Walzl A., Unger C., Rosner M., Krupitza G., Hengstschläger M., Dolznig H. (2013). In vitro cell migration and invasion assays. Mutat. Res. Rev. Mutat. Res..

[35] Ortiz A., Gui J., Zahedi F., Yu P., Cho C., Bhattacharya S., Carbone C.J., Yu Q., Katlinski K.V., Katlinskaya Y.V. (2019). An Interferon-Driven Oxysterol-Based Defense against Tumor-Derived Extracellular Vesicles. Cancer Cell.

[36] Whitehead B., Wu L., Hvam M.L., Aslan H., Dong M., Dyrskjøt L., Ostenfeld M.S., Moghimi S.M., Howard K.A. (2015). Tumour exosomes display differential mechanical and complement activation properties dependent on malignant state: Implications in endothelial leakiness. J. Extracell Vesicles.

[37] Ghoshal A., Rodrigues L.C., Gowda C.P., Elcheva I.A., Liu Z., Abraham T., Spiegelman V.S. (2019). Extracellular vesicle-dependent effect of RNA-binding protein IGF2BP1 on melanoma metastasis. Oncogene.

[38] Xiao D., Ohlendorf J., Chen Y., Taylor D.D., Rai S.N., Waigel S., Zacharias W., Hao H., McMasters K.M. (2012). Identifying mRNA, MicroRNA and Protein Profiles of Melanoma Exosomes. PLoS ONE.

[39] Mannavola F., Tucci M., Felici C., Passarelli A., D’Oronzo S. (2019). Tumor-derived exosomes promote the in vitro osteotropism of melanoma cells by activating the SDF-1/CXCR4/CXCR7 axis. J. Transl. Med..

[40] Lazar I., Clement E., Dauvillier S., Milhas D., Ducoux-Petit M., LeGonidec S., Moro C., Soldan V., Dalle S., Balor S. (2016). Adipocyte Exosomes Promote Melanoma Aggressiveness through Fatty Acid Oxidation: A Novel Mechanism Linking Obesity and Cancer. Cancer Res..

[41] Plebanek M.P., Angeloni N.L., Vinokour E., Li J., Henkin A., Martinez-Marin D., Filleur S., Bhowmick R., Henkin J., Miller S.D. (2017). Pre-metastatic cancer exosomes induce immune surveillance by patrolling monocytes at the metastatic niche. Nat. Commun..

[42] Tung K.H., Ernstoff M.S., Allen C., Shu S. (2019). A Review of Exosomes and their Role in The Tumor Microenvironment and Host-Tumor "Macroenvironment". J. Immunol. Sci..

[43] Logozzi M., Mizzoni D., Angelini D.F., Di Raimo R., Falchi M., Battistini L., Fais S. (2018). Microenvironmental pH and Exosome Levels Interplay in Human Cancer Cell Lines of Different Histotypes. Cancers.

[44] Kim H., Lee S., Shin E., Seong K.M., Jin Y.W., Youn H., Youn B. (2020). The Emerging Roles of Exosomes as EMT Regulators in Cancer. Cells.

[45] Gener Lahav T., Adler O., Zait Y., Shani O., Amer M., Doron H., Abramovitz L., Yofe I., Cohen N., Erez N. (2019). Melanoma-derived extracellular vesicles instigate proinflammatory signaling in the metastatic microenvironment. Int. J. Cancer.

[46] Shu S.L., Yang Y., Allen C.L., Maguire O., Minderman H., Sen A., Ciesielski M.J., Collins K.A., Bush P.J., Singh P. (2018). Metabolic reprogramming of stromal fibroblasts by melanoma exosome microRNA favours a pre-metastatic microenvironment. Sci. Rep..

[47] Siveen K.S., Raza A., Ahmed E.I., Khan A.Q., Prabhu K.S., Kuttikrishnan S., Mateo J.M., Zayed H., Rasul K., Azizi F. (2019). The Role of Extracellular Vesicles as Modulators of the Tumor Microenvironment, Metastasis and Drug Resistance in Colorectal Cancer. Cancers.

[48] Tian X., Shen H., Li Z., Wang T., Wang S. (2019). Tumor-derived exosomes, myeloid-derived suppressor cells, and tumor microenvironment. J. Hematol. Oncol..

[49] Whiteside T. (2008). The tumor microenvironment and its role in promoting tumor growth. Oncogene.

[50] Tucci M., Mannavola F., Passarelli A., Stucci L.S., Cives M., Silvestris F. (2018). Exosomes in melanoma: A role in tumor progression, metastasis and impaired immune system activity. Oncotarget.

[51] Düchler M., Czernek L., Peczek L., Cypryk W., Sztiller-Sikorska M., Czyz M. (2019). Melanoma-Derived Extracellular Vesicles Bear the Potential for the Induction of Antigen-Specific Tolerance. Cells.

[52] Isola L.A., Chen S. (2017). Exosomes: The messengers of health and disease. Curr. Neuropharmacol..

[53] Hood J.L., San R.S., Wickline S.A. (2011). Exosomes released by melanoma cells prepare sentinel lymph nodes for tumor metastasis. Cancer Res..

[54] Passarelli A., Mannavola F., Stucci L.S., Tucci M., Silvestris F. (2017). Immune system and melanoma biology: A balance between immunosurveillance and immune escape. Oncotarget.

[55] Ventola C.L. (2017). Cancer Immunotherapy, Part 1: Current Strategies and Agents. Pharm. Ther..

[56] Wieckowski E.U., Visus C., Szajnik M., Szczepanski M.J., Storkus W.J., Whiteside T.L. (2009). Tumor-derived microvesicles promote regulatory T cell expansion and induce apoptosis in tumor-reactive activated CD8+ T lymphocytes. J. Immunol..

[57] Zhou J., Yang Y., Wang W., Zhang Y., Chen Z., Hao C., Zhang J. (2018). Melanoma-released exosomes directly activate the mitochondrial apoptotic pathway of CD4+ T cells through their microRNA cargo. Exp. Cell Res..

[58] Yin Y., Cai X., Chen X., Liang H., Zhang Y., Li J., Wang Z., Chen X., Zhang W., Yokoyama S. (2014). Tumor-secreted miR-214 induces regulatory T cells: A major link between immune evasion and tumor growth. Cell Res..

[59] Hiltbrunner S., Larssen P., Eldh M., Martinez-Bravo M.J., Wagner A.K., Karlsson M.C., Gabrielsson S. (2016). Exosomal cancer immunotherapy is independent of MHC molecules on exosomes. Oncotarget.

[60] Chen G., Huang A.C., Zhang W., Zhang G., Wu M., Xu W., Yu Z., Yang J., Wang B., Sun H. (2018). Exosomal PD-L1 contributes to immunosuppression and is associated with anti-PD-1 response. Nature.

[61] Wu Y., Deng W., McGinley E.C., Klinke D.J. (2017). Melanoma exosomes deliver a complex biological payload that upregulates PTPN11 to suppress T lymphocyte function. J. Extracell Vesicles.

[62] Cheng L., Wang Y., Huang L. (2017). Exosomes from M1-Polarized Macrophages Potentiate the Cancer Vaccine by Creating a Pro-inflammatory Microenvironment in the Lymph Node. Mol. Ther..

[63] Sharma P., Diergaarde B., Ferrone S., Kirkwood J.M., Whiteside T.L. (2020). Melanoma cell-derived exosomes in plasma of melanoma patients suppress functions of immune effector cells. Sci. Rep.-UK.

[64] Vella L.J., Behren A., Coleman B., Greening D.W., Hill A.F., Cebon J. (2017). Intercellular Resistance to BRAF Inhibition Can Be Mediated by Extracellular Vesicle–Associated PDGFRβ. Neoplasia.

[65] Cesi G., Philippidou D., Kozar I., Kim Y.J., Bernardin F., Van Niel G., Wienecke-Baldacchino A., Felten P., Letellier E., Dengler S. (2018). A new ALK isoform transported by extracellular vesicles confers drug resistance to melanoma cells. Mol. Cancer.

[66] Vu L.T., Gong J., Pham T.T., Kim Y., Le M.T.N. (2020). microRNA exchange via extracellular vesicles in cancer. Cell Proliferat..

[67] Greenberg E., Hershkovitz L., Itzhaki O., Hajdu S., Nemlich Y., Ortenberg R., Gefen N., Edry L., Modai S., Keisari Y. (2011). Regulation of cancer aggressive features in melanoma cells by microRNAs. PLoS ONE.

[68] Gajos-Michniewicz A., Czyz M. (2019). Role of miRNAs in melanoma metastasis. Cancers.

[69] Gajos-Michniewicz A., Duechler M., Czyz M. (2014). MiRNA in melanoma-derived exosomes. Cancer Lett..

[70] Svedman F.C., Lohcharoenkal W., Bottai M., Brage S.E., Sonkoly E., Hansson J., Pivarcsi A., Eriksson H. (2018). Extracellular microvesicle microRNAs as predictive biomarkers for targeted therapy in metastastic cutaneous malignant melanoma. PLoS ONE.

[71] Lee J.H., Dindorf J., Eberhardt M., Lai X., Ostalecki C., Koliha N., Gross S., Blume K., Bruns H., Wild S. (2019). Innate extracellular vesicles from melanoma patients suppress β-catenin in tumor cells by miRNA-34a. Life Sci. Alliance.

[72] Dror S., Sander L., Schwartz H., Sheinboim D., Barzilai A., Dishon Y., Apcher S., Golan T., Greenberger S., Barshack I. (2016). Melanoma miRNA trafficking controls tumour primary niche formation. Nat. Cell Biol..

[73] Felicetti F., De Feo A., Coscia C., Puglisi R., Pedini F., Pasquini L., Bellenghi M., Errico M.C., Pagani E., Carè A. (2016). Exosome-mediated transfer of miR-222 is sufficient to increase tumor malignancy in melanoma. J. Transl. Med..

[74] House I.G., Petley E.V., Beavis P.A. (2018). Tumor-derived exosomes modulate T cell function through transfer of RNA. FEBS J..

[75] Li J., Chen J., Wang S., Li P., Zheng C., Zhou X., Tao Y., Chen X., Sun L., Wang A. (2019). Blockage of transferred exosome-shuttled miR-494 inhibits melanoma growth and metastasis. J. Cell Physiol..

[76] Huber V., Vallacchi V., Fleming V., Hu X., Cova A., Dugo M., Shahaj E., Sulsenti R., Vergani E., Filipazzi P. (2018). Tumor-derived microRNAs induce myeloid suppressor cells and predict immunotherapy resistance in melanoma. J. Clin. Invest..

[77] Luan W., Ding Y., Xi H., Ruan H., Lu F., Ma S., Wang J. (2021). Exosomal miR-106b-5p derived from melanoma cell promotes primary melanocytes epithelial-mesenchymal transition through targeting EphA4. J.Exp. Clin. Cancer Res..

[78] Dar A.A., Majid S., de Semir D., Nosrati M., Bezrookove V., Kashani-Sabet M. (2011). miRNA-205 Suppresses Melanoma Cell Proliferation and Induces Senescence via Regulation of E2F1 Protein*. J. Biol. Chem..

[79] Segura M.F., Hanniford D., Menendez S., Reavie L., Zou X., Alvarez-Diaz S., Zakrzewski J., Blochin E., Rose A., Bogunovic D. (2009). Aberrant miR-182 expression promotes melanoma metastasis by repressing FOXO3 and microphthalmia-associated transcription factor. P. Natl. A. Sci. Bel. Agr..

[80] Shull A.Y., Latham-Schwark A., Ramasamy P., Leskoske K., Oroian D., Birtwistle M.R., Buckhaults P.J. (2012). Novel Somatic Mutations to PI3K Pathway Genes in Metastatic Melanoma. PLoS ONE.

[81] Shi Q., He Q., Wei J. (2018). MicroRNA-342 prohibits proliferation and invasion of melanoma cells by directly targeting Zinc-finger E-box binding homeobox 1. Oncol. Res..

[82] Wang J., Wuethrich A., Sina A.A., Lane R.E., Lin L.L., Wang Y., Cebon J., Behren A., Trau M. (2020). Tracking extracellular vesicle phenotypic changes enables treatment monitoring in melanoma. Sci. Adv..

[83] Melling G.E., Carollo E., Conlon R., Simpson J.C., Carter D.R.F. (2019). The Challenges and Possibilities of Extracellular Vesicles as Therapeutic Vehicles. Eur. J. Pharm. Biopharm..

[84] Willis G.R., Kourembanas S., Mitsialis S.A. (2017). Therapeutic Applications of Extracellular Vesicles: Perspectives from Newborn Medicine. Methods Mol. Biol..

[85] Wiklander O.P.B., Brennan M.Á., Lötvall J., Breakefield X.O., EL Andaloussi S. (2019). Advances in therapeutic applications of extracellular vesicles. Sci. Transl. Med..

[86] Luke J.J., Flaherty K.T., Ribas A., Long G.V. (2017). Targeted agents and immunotherapies: Optimizing outcomes in melanoma. Nat. Rev. Clin. Oncol..

[87] Abdouh M., Floris M., Gao Z.H., Arena V., Arena M., Arena G.O. (2019). Colorectal cancer-derived extracellular vesicles induce transformation of fibroblasts into colon carcinoma cells. J. Exp. Clin. Cancer Res..

[88] Samuel M., Fonseka P., Sanwlani R., Gangoda L., Chee S.H., Keerthikumar S., Spurling A., Chitti S.V., Zanker D., Ang C.-S. (2021). Oral administration of bovine milk-derived extracellular vesicles induces senescence in the primary tumor but accelerates cancer metastasis. Nat. Commun..

[89] Macklin R., Wang H., Loo D., Martin S., Cumming A., Cai N., Lane R., Ponce N.S., Topkas E., Inder K. (2016). Extracellular vesicles secreted by highly metastatic clonal variants of osteosarcoma preferentially localize to the lungs and induce metastatic behaviour in poorly metastatic clones. Oncotarget.

[90] Serpe C., Monaco L., Relucenti M., Iovino L., Familiari P., Scavizzi F., Raspa M., Familiari G., Civiero L., D’Agnano I. (2021). Microglia-Derived Small Extracellular Vesicles Reduce Glioma Growth by Modifying Tumor Cell Metabolism and Enhancing Glutamate Clearance through miR-124. Cells.

[91] Iessi E., Logozzi M., Lugini L., Azzarito T., Federici C., Spugnini E.P., Mizzoni D., Di Raimo R., Angelini D.F., Battistini L. (2017). Acridine Orange/exosomes increase the delivery and the effectiveness of Acridine Orange in human melanoma cells: A new prototype for theranostics of tumors. J. Enzyme Inhib. Med. Chem..

[92] Lin Q., Qu M., Zhou B., Patra H.K., Sun Z., Luo Q., Yang W., Wu Y., Zhang Y., Li L. (2019). Exosome-like nanoplatform modified with targeting ligand improves anti-cancer and anti-inflammation effects of imperialine. J. Control. Release..

[93] Tarasov V.V., Svistunov A.A., Chubarev V.N., Dostdar S.A., Sokolov A.V., Brzecka A., Sukocheva O., Neganova M.E., Klochkov S.G., Somasundaram S.G. (2019). Extracellular vesicles in cancer nanomedicine. Semin. Cancer Biol..

[94] Munagala R., Aqil F., Jeyabalan J., Gupta R.C. (2016). Bovine milk-derived exosomes for drug delivery. Cancer Lett..

[95] Koyama Y., Ito T., Hasegawa A., Eriguchi M., Inaba T., Ushigusa T., Sugiura K. (2016). Exosomes derived from tumor cells genetically modified to express Mycobacterium tuberculosis antigen: A novel vaccine for cancer therapy. Biotechnol. Lett..

[96] Erkan E.P., Saydam N., Chen C.C., Saydam O. (2019). Extracellular vesicles as carriers of suicide mRNA and/or protein in cancer therapy. Methods Mol. Biol..

[97] Chao O.S., Chang T.C., Di Bella M.A., Alessandro R., Anzanello F., Rappa G., Goodman O.B., Lorico A. (2017). The HDAC6 Inhibitor Tubacin Induces Release of CD133(+) Extracellular Vesicles From Cancer Cells. J. Cell Biochem..

